# On the Education of Children Predisposed to Insanity

**Published:** 1848-10-01

**Authors:** 


### THE JOURNAL

OF

### PSYCHOLOGICAL MEDICINE

AND

### MENTAL PATHOLOGY.

OCTOBER 1, 1848.

Over

### Analytical Reviews.

ART. I.—*The use of the Body in relation to the Mind.* By GEORGE MOORE, M.D., Member of the Royal College of Physicians. Second Edition. Longman, 1847. 8vo, pp. 433.

*Practical Hints on the Moral, Mental, and Physical Training of Girls at School.* By MADAME DE WAHL. Parker, 1847. Small 8vo, pp. 190.

"*Pourquoi,*" said the Cardinal Polignac, quoted by Gall,—"*pourquoi des hommes très vicieux, pour qui le crime à des delices, et qui ne se croient pas criminels, se repentiraient-ils?*" Read the biographies of all the tyrants who have ever yet desolated the earth, and say, if you can discover one who voluntarily renounced his crimes ere death or public vengeance had cut him off from fellowship with mankind. For the study of psychology teaches us, that gross delinquents are for the most part inaccessible to pity or remorse, and that the moral sense, abandoned to its own blind caprice, rarely fails in warping the judgment, and misguiding the steps, not only in the jocund hour of youth, but, what is still worse, in the more mature season of manhood, ambition and strength. In persons of feeble organization and imbecile temperament, the perception of good and evil lies dormant or inert, suffering the wild fire of passion to flame and flicker over their slumbering energies with its alluring, seductive, and fatal fascinations. Self-indulgence covers the broad way to destruction; and in the condign felon or the hopeless maniac we may detect the first neglected germ of all his miseries—viz., *the want of self-control.* The extent of depravity to which this single moral deficiency is sure to lead, would be incredible, were it not but too well attested both by history and our own common experience. The perfidious and cruel Gabrino-Fundulo, condemned to lose his head on account of his atrocious crimes, fiercely confessed, that the only thing he ever repented of, was not having precipitated Pope John XXIII. and the Emperor Sigismond from the top of the tower of Cremona, when they adventurously ascended that dangerous pinnacle together with him;* and Dion Cassius† informs us, that what added to the horror of a plague that broke out at Rome and carried off 2000 persons in a day, was the desperate wickedness of certain individuals who went about pricking others with poisoned needles with the view of causing their death; and that when some of these culprits were arrested and put to extreme punishment, they confessed, that for the sake of a bribe they had been induced to perpetrate this horrible deed.

The wretch devoid of conscience is of course morally defunct; but we must never forget that conscience is a relative, not an absolute term, and that, like every other faculty, it requires education, direction, and discipline. The cannibal, who first fights and kills and then eats his enemy, pretends to the *mens sibi conscia recti* just as confidently as the soldier who receives a baton, a ribbon, a title, or a star as the reward of merit and the distinguished token of his military prowess. For the standard of rectitude ascends and declines with the shifting habits of society, the prevailing notions of the day, and the spirit of the age in which we play our part. The natural conscience, so proper to our hearts, is superseded by an artificial one (the product of civilization or barbarism), which comes and goes with the fashion of the world, glimmering in the morals of Seneca, the odes of Horace, the coterie of *un roi-philosophe* such as Frederic of Prussia, the myths of ancient Egypt, the strange astrology of the Chaldæan Magi, and the antique, gorgeous repasts that have long since loomed and vanished in perspective along the halls of Old Belus. Such and so various are the destinies of mankind, that we are almost tempted to look on and disregard passing events with as much indifference and contempt as we do the racket-ball that leaps from the player's hand and rebounds only to be struck back by him again.

But the necessity of living confines us to our post, and forces us to observe and attend with the deepest interest. Every age has felt and acknowledged the difficulty of solving the problem of EDUCATION. Each family carries within its own bosom the fruits of a good or an evil one. Lycurgus and Solon, convinced, like all other legislators, that the fate of empires depended on the education of youth, exhausted their best energies in designing a mode of teaching which should at the same time strengthen the body and elevate the mind to the highest degree of physical and moral perfection. The divine legation of Moses, under the sanction and guidance of a far more authentic archetype than theirs, was primarily directed to the attainment of this same mighty object. Read the canons of the councils of the church throughout the middle ages, and you will find the same theme pervading them all—namely, the repeated inculcation of strict moral habits as the surest correction of error, delinquency, and vice. Survey every country in Europe, not even excepting Great Britain herself, and see how the hands of each government are shackled by a population, *vicious* because they are *untaught*.

* Gall sur les fonctions du Cerveau, tome v. pp. 284—294.

† Hist. Roman., lxvii. 11; lxxii. 14; quoted by Littré, Œuvres d'Hippocrate, traduction nouvelle, Paris, 1846, tome v. p. 68.

and *ungovernable* because they are *ignorant.** Enter the workhouse or the asylum and count the numbers whose brains have been cracked by the load of life which they were never taught how to carry with ease and safety to themselves. Obtain admittance into the glittering mansions that adorn our cities or our parks, and behind their sumptuous hangings you will meet a spectre with the sign and seal on its front of woeful self-indulgence—manhood sapped in its prime, talents wasted at their source, and the warmest affections cankered at their core! The chance medley of the world everywhere discloses scenes of depravity, ignorance, vice, and want, which are only the more disgusting among the lower orders because they are not glazed over with the semblance of virtue so pleasing in the saloons of the wealthier and more influential classes of society. Lust, which is, as it were, a sculptured idol of gold to be adored or adulated by the rich, lurks like a foul fiend within the hovels of penury and dirt. And the mind and the moral being, where are they? Where is the life to come, the present hour, peace, contentment, wisdom, and the happiness of house and home? Are they the fictions of the poet, or the idle visions of a religion that has no existence save in the unreal mockery of a sublime ideal?

One of the surest consequences of self-indulgence is the loss of mind, account for it as we please on physiological or metaphysical principles;

* Thirty-thousand destitute children disgrace the metropolis of Great Britain. Out of a given number of 1600 young persons,—162, one-tenth of the whole, confessed that they had been frequently in prison; 116 had run away from home; 170 slept in lodging-houses which are the nests of everything that the human mind could conceive of abominable; 253 avowed that they lived by begging; 216 had no shoes or stockings; 280 had no caps, hats, or bonnets; 101 had no body linen; 249 never slept, or had no recollection of ever having slept in a bed; 68 were the children of convicts; 125 had step-mothers, to whom might be traced much of the misery that drove them to the commission of crime; and 306 had lost either one or both parents.—*Parliamentary Reports, Lord Ashley's Speech on Emigration and Ragged Schools, June 5th*, 1848.

The facts, at least such as have come within our own knowledge, rather tend to demonstrate that spirit-drinking, debauchery, excesses of all sorts in the parents, and occasionally the debility of privation and the abuse of mercurial medicine, have been the principal causes of the lamentable increase of diseases of the brain in children; but these are rendered more intensely mischievous to the offspring by the misery of mind which accompanies bad habits, and depresses the moral being into reckless despondency.—*Dr. Moore, op. cit. ut supra*, p. 98.

The serious evils inseparable from dark and filthy dwellings, such as abound in all our large cities, are incalculable. It is easy to conceive the physical evils of such crowding, want of ventilation, and filth. It is equally obvious, that where decency and decorum are violated, and the most corrupting and revolting associations are not only promoted but rendered unavoidable, the moral evils which result from them must be enormous; and it is no marvel that such quarters are the nurseries of the criminals who fill our jails.—*Means of Promoting and Preserving Health. By T. Hodgkin, M.D. 2nd edit.*, 1841, p. 38.

We observed to Captain Stuart, the superintendent of the police at Edinburgh, in one inspection of the Wynds, that life appeared of little value, and was likely to be held cheap in such spots. He stated, in answer, that a short time ago, a man had been executed for the murder of his wife, in a fit of passion, in the very room we had accidentally entered, and where we were led to make the observation. At a short distance from that spot, and amidst others of this class of habitation, were those which had been the scenes of the murders of Burke and Hare. The working-classes in these districts were equally marked by the abandonment of every civil or social regulation.—*Mr. Chadwick on the Labouring Population, quoted by William Kebbell, M.D., on the Diseases of Towns. Brighton*, 1848, p. 94.

for there is nothing so true as that the *continual indulgence of the appetites leads to the premature extinction of the reason.* Those who go down to the grave with clear minds, good eyesight, sound teeth, unparalysed limbs, and placid tempers, are the self-denying and the temperate; the modest and the retired. It is as our days are spent that our years are told; and the virtuous demeanour that imparts such a charm to the venerable patriarch is, independent of natural vigour, the last flower of the seed sown in the early twilight of his days.

Now, Dr. Moore and Madame de Wahl, both of whose works we have placed at the head of this article, have seriously taken up the subject of mental culture, at opposite points of view indeed, but with equal sincerity, piety, and zeal. The distinguishing feature of Madame de Wahl's interesting brochure is that of good sense, joined with that nice perception of propriety so peculiar to a lady's writings. Our own notions are there easily expressed for us upon a subject on which we acknowledge that our pen would be by far too heavy and laborious, if not altogether inefficient; and we thank her for her pains. Dr. Moore's work, which is a medical essay addressed *ad laicos,* considers the grand question of education on its physiological and moral basis, and is, therefore, the more discursive and philosophic of the two. Without quoting verbatim from either, we shall avail ourselves of the materials which they have both so happily placed at our disposal.

The chief mark at which each of these writers aims is that of self-control. As human beings, we are not mere spirit, for our soul is united to a body, not like that of the lower animals governed by instinct, but instigated by active feelings that are perpetually revolting and making war against the jealous supremacy of the soul. By the aid of religious restraints, moral discipline, or legal penalties, this ruling power is assisted in maintaining the ascendant; and, for the most part, on common occasions, is enabled to direct its very awkward companion, the body, to the best and surest ends. For, so long as we are refreshed by sleep, nourished by food, entertained by conversation, and amused by seeing, walking, and acting, life proceeds peaceably enough along its beaten and accustomed path; but when pride, covetousness, lust, anger, gluttony, envy, or sloth, are engendered within the breast, then kindles and breaks forth the strife of passion, which all the philosophers in the world, from Plato down to Kant, have owned themselves weaponless and defenceless in fighting against. It is a commotion that overthrows the noblest faculties of man, and ruthlessly tramples on the heart. For how can you expect, that in one, addicted to the grosser pleasures, the heart should go on beating with the usual placidity of health? or, that the nervous system should respond with accurate vibrations throughout the unsettled tracery of its delicate fibres? It is impossible; and, in exact proportion to the assaults of ungovernable passion, does the intellect, shaken to its centre, threaten to decline. Mania may be implanted in its germ, and the passionate child might, unchecked or uncorrected, become a madman or a fool.

Insanity is not the growth of a day or a week; frequently it is the product of years, the germ being generated and the seed often sown, in early life. The experienced psychologist can trace back its origin to a very early date, when it assumed no other form than that of caprice, (a very suspicious symptom at all times,) self-will, ungovernable passion, want of self-control, a propensity to lie, to steal, to drink, stimulation to excess, a tendency to excessive dislike—to motiveless dislike—a pleasure in acts of cruelty, irritability, a temper irascible only on a single point (*fœnum habet in cornu*), and a peculiar expression of the eye which it is not possible to describe by the pencil or the pen, and yet is so very significant. We have predicted mania in individuals whose gait and habits have alone betrayed the lurking malady to us. Some children are born idiotic, and even in the nurse's arms there is an oddity of manner that bespeaks the lack of ordinary intelligence. As they grow up, their passions are always violent, and not amenable to reason; or they display their angry feelings without a cause, or give vent to them out of all proportion to the cause. During the development of their minds, particular ideas, incompatible with the comfort, order, and accustomed habits of those about them, are evinced and pertinaciously acted upon. Selfishness is the ruling motive; and spite, malignity, taciturnity, and dogged perverseness are manifested in return for the mildest check placed upon their unruliness. Teaching, according to the usual routine of schools, is out of the question;—the boy runs away, regardless of disgrace and shame, or the girl heads a party and takes the greatest delight in trying to corrupt the rest. Their after-lives are in accordance with their youth; and the misguided or head-strong woman abandons herself to excesses, while the perverse man excommunicates himself from society by a wanton dereliction of duty, totally at variance with the received modes of society in which they had been respectively brought up. It would be easy to account for these extravagances upon moral grounds alone, or, phrenologically speaking, according to particular organizations of the centro-spinal system; and, doubtless, over-indulgence on the part of parents, and defects on the side of nature, are very powerful and common causes. Poetry, music, novel-reading (*illuminism*), and dissipated pleasures, operate very largely in promoting the development of deleterious passions, by exciting the imagination and the finer sympathies of our nature to a morbid degree of exaltation, and substituting an ideal for the ordinary standard of excellence. Some of the eccentricities that tarnish the lustre of names famous in history, may be imputed to sources such as these. Cambyses, after burying a dozen Persians alive, as Herodotus tells us, in Thalia, shot the son of Praxaspes through the heart with an arrow, on purpose to prove his own sanity, so seriously called into question by his ablest subjects. The ancients affirmed that Orestes was struck with madness for killing his mother; Œdipus, for a similar crime, and Ajax Oileus, for violating the sanctity of a temple. Dolabella, Cicero's worthless son-in-law, who demolished the altar erected to the *latreia* of Cæsar, subsequently miscarried in all his public undertakings, and at last terminated his existence with his own hands. This was the sacred disease of the ancients, which gave rise to the hackneyed line, "*Quem Jupiter vult perdere prius dementat.*" There can, likewise, be no doubt that peculiar conformations of the body indicate or produce peculiarities of the mind and temper, so that a giant is popularly stigmatized as big and stupid, while dwarfs are generally looked down upon as conceited and spiteful. Distortions of the spine are proverbial for the irritability of the brain, the vivacity of thought, and the biting sarcastic humour incidental to their deformity, such as every one is familiar with in the persons of Pope and Æsop. Large foreheads are proper to philosophers; eyes wide apart, to draftsmen; rotund temporal fossæ to architects and misers; and projecting orbitar ridges to calculators, chronologists, and the lovers of order and colour. Thick lips betray the gourmand and the debauchee, aquiline noses signify even tempers and clever understandings, thin lips insinuate a vindictive disposition, and projecting chins point out either buffoonery, or its direct opposite, mental ascendancy and clear common sense. Byron, Hunter, and Newton, have each of them the organic development essential to the manifestation of their personal talents; and the historian, Gibbon, with his prominent eyeball and voluminous cranium, may be profitably compared with the retreating forehead of Robespierre, Nero, and the profile of the Dutch adult idiot portrayed by Gall. Hereditary taint is another fruitful source of moral and intellectual eccentricities; and so is scrofula, that prolific origin of all that is imperfect, excessive, ecstatic, obstinate, and incurable in the physical history of man. Crooked spines, gout, asthma, phthisis, mania, imbecility of mind or body, short lives, effæte offspring, and an existence scarcely worth the having, fill up the weary catalogue of its daily—nay, of its hourly lamentations. See Essays on Scrofula, by W. Ring, M.D., Cantab., Brighton Med. Gaz. 1847.

All these are causes of mental derangement beginning very early in life, almost as early as the cradle, and eventually producing a class of diseases as difficult to manage as they are dangerous in their tendencies. Treatises have been written upon them, and plans of treatment have been devised, reflecting the greatest credit on the benevolence and sagacity of their authors. The dead-house has yielded its stock of scanty information. The pharmacopæia has been searched for remedies, which have been vaunted for a time, found ineffectual, and then laid aside and forgotten. Religious training has, of course, not been omitted, and the Scriptures have been quoted for the thousandth and first time, but, as usual, all in vain. Physiology has not been left out of the inquiry, nor even dry statistics, so much in vogue at present, with its tiresome list of unimaginative numerals. All has been tried, and hitherto in a great measure without success, because, perhaps, no one has gone back to the beginning, and acted on the adage of a by-gone generation, that prevention is better than cure. It might be affirmed, that, if a great deal too much has been attempted, a great deal more has been left undone in the prevention of insanity. This omission is in the nursery—at that season and in those hours when nobody but the nurse and the mother are in attendance on the patient, and when the malady is as yet in its latent state, invisible to unskilled eyes. This is the point at which we must begin in order to effect a radical cure, nor can we justly hope to succeed, even at this timely moment, until a subsequent generation of mothers shall prove themselves to be more enlightened in the art of preserving both the mental and bodily health of their children than we can allow the present "*heads of houses*" the honour of being fairly entitled to.

As it is not our intention to enter on all the minutiæ of hygiene,* so ably prescribed in works written expressly on this subject, and, indeed, so satisfactorily dwelt upon in the two essays at the head of this article, especially that of Madame de Wahl (an earnest of better things), we will consider one point only, and this is the power of self-control. Taking it for granted, that, in the greater number of cases, no organic change has actually occurred, and that nothing else than a morbid excitement prevails within the sensorium, we are confident that not only actual disease itself will be warded off, but that a share of happiness and buoyancy of animal spirits can be attained by a cultivation of the habit of self-control and self-respect, early acquired, which none but those who are intimately acquainted with the lives of children,† and the protracted history of mania, in its manifold shapes and phases, will ever feel themselves inclined to assent to. By self-control, we do not mean the harsh government of a child—the rod, the menacing mien, and the drum-head court-martial of a little garrison. Nothing of the kind. The child should be early taught that there are some things which it may, and others which it may not do, and it should be shown and given to understand that the permission is as conducive to its happiness and comfort in the one case as the prohibition is in the other, and this regulation should be made with united mildness and firmness, and without, for a single moment, interrupting the favourite game of play, the merry laugh, and the frolic and fun, which, alas! sooner or latter passes away with the bright sunshine belonging to the morning of life. Particular cases will require particular management. The tendency to avarice‡ may be counterbalanced by the superinduced habit of liberality; sloth by that of activity; and native haughtiness and pride by daily inducements to delight in the practice of humility. Children are naturally curious, eager after information, the why and the wherefore of everything, and what they have once learned they never forget. With this happy discipline, which will render learning a pastime rather than a task, should be conjoined *a course of instruction on the realities of life.* Everything should be real—nothing should be covered or glossed over,

* There is a very interesting work entitled *Mental Hygiene, or an examination of the intellect and passions, designed to illustrate their influence on health and the duration of life. By William Sweetser, M.D., New York,* 1843. It is cleverly composed, and the style, if not everywhere quite correct, is always lucid. Its author is evidently a diligent student and a thoughtful medical man.

† Those who have, like the writer, been much in the nursery and among the lower orders, will scarcely have failed to remark a strong resemblance between children, ignorant persons, and idiots. The idiot is an absolutely ignorant child, the ignorant person is a conditional idiot, and the child is an ignorant postulant. Hence the difficulty of guiding young persons, and of governing ignorant masses of the population, whose motives are selfish, imprudent, impolitic, misdirected, and headstrong.

‡ A young man, who had grown weary of waiting for the death of a wealthy relative, whose gold he was to inherit, at length came into possession of his long wished for treasures. But very soon afterwards he was seized with a serious illness, and his physician, foreseeing his end approaching, gave him to understand that if he had any affairs to settle in this world, the sooner he did so the better, as he had no time to lose. "What!" exclaimed the irritated heir, starting up with the dew of his last agony on his brow; "what!—I die?—impossible!—I have not had time to enjoy my property yet!" So saying, he fell back on his pillow, a corpse.

and made to appear like what it is not. This reality should extend to the dress and the behaviour, which ought to be the same in the nursery as it is in the parlour. Their daily habits ought to be as real as their speech, and the reality of their speech will offend no one if it spring from habits that are really virtuous. There can be no virtue where there is no reality, and no religion where there is not virtue. And what is reality, but truth? and what is unreality or pretence, but falsehood or double-mindedness? and what is mania, but a false or unreal condition, which may end in a permanently disorganized state of the once really healthy mind? The actual madman knows no difference between truth and falsehood, between the positive verities of nature and the negative idealities of his own morbid dream—out of which, perhaps, he never awakes except in the life to come. O, if men but knew the inestimable value of truth, and the ultimate horror, to say nothing of the bad policy of a lie, whether it be an acted or a spoken one!

If there is any other virtue besides self-control which exerts a beneficial and salubrious effect on the functions of the living organism, it is that of humility. The humble minded person (actual disease apart) can scarcely become insane. The chances and changes of this mortal life flow over without buffeting him with its waves. No position in the world is to him either high or low; for he knows that every affair must come to a close, and that the longest life is short. He respects mankind as he wishes to be respected, and in the imperfections of others he beholds the admonitory reflection of himself.

We have been speaking of those with apparently healthly constitutions in whom insanity is surreptitiously produced as a consequence of moral mismanagement. But we have seldom seen such individuals as are decidedly predisposed to mania, who do not exhibit some organic malformations of the frame, especially of the nervous system or its appurtenances, too visible to be overlooked—some strong hereditary taint or inveterate scrofulous diathesis. Some children, thus predisposed, are deaf, others short-sighted, or the subjects of lippitudo or incipient amaurosis; or they are disfigured by glandular enlargements, or a short leg, or a strumous hip-joint, or a club-foot, or nævi, or encysted tumours of the scalp, or crooked squab features with an acute facial angle, and a low slanting forehead. The vertex is often prolongated, and the hair, straight and stiff, radiates from the crown, and falls over the head like a night-cap. The eye tells a great deal—its movements are restless or drowsy, and the upper eyelid hangs down half over the globe, or else from sudden emotion is spasmodically elevated, showing the white of the eye all the way round the iris. There is a spasm or twitch of a leg or an arm, and the chin is thrown up, and in moments of gratification, chiefly sensual, the smile of pleasure passes off into the grin of the *risus sardonicus*. The mouth is generally open, the under lip pendant, and it often slavers. In general the stature is short, with a long back, stunted legs, and long arms. All these signs may not exist in conjunction, nor any one of them in so very marked a form as in that above delineated; but that they do exist in a more or less modified degree, the practised eye will not be slow in perceiving, nor in deducing the legitimate inferences.

It is obvious, that in cases of this description, ordinary tuition is out of the question. Much must depend on particular indications, and be adapted according to the judgment of the practitioner, and the local advantages of the patient. A situation high and dry, in the country, apart from the noise and dense atmosphere of a city, is indispensable. Plenty of wholesome and unstimulating food,* early hours of rising and retiring to rest, ablution, and personal cleanliness, are among the first requisites towards recovery, amendment, or an amelioration of so unpromising a condition. As to moral superintendence, the intellect must be cultivated less sedulously than the feelings; and the feelings must be cherished and won over, as much as possible, to sociality and a cheerful intercourse with others. Peculiar propensities or antipathies must be diverted rather than checked, and drawn aside from an improper object and carefully directed towards a more suitable one. Manual employment will be found of the greatest service, for there is nothing so conducive to health, and so invigorating to the mind, as manual labour. We would especially recommend such an occupation as is concerned in rural pursuits—the garden, the field, or even following the plough with a proper attendant in fair weather, and, in winter, sweeping, dusting, arranging the furniture, &c.—taking part, in short, in some of the daily business of the household affairs. According to the class in life, the occupation may be more or less menial; but, whether menial or not, the pursuit must be an active one, and the hands must be exercised more than the head. Habit is everything—the habit of being tidy, orderly, harmlessly engaged, and of having the time filled up with useful trifles; so that what is begun as a habit shall be persisted in because it is habitual. Education, which is nothing more than a repeated drill, commences with the body rather than with the mind; and the experienced teacher knows that, in the best constituted intellects, the first difficulty to be overcome is that of rendering the body subservient to the will. We are the creatures of habit, and use is second nature. It is upon this principle we must act, and our main endeavour must be to counteract idiotic or maniacal tendencies by counterfeiting the practice of common sense till it become habitual and natural. Nor does the good effect of this kind of training terminate with the mere habit thus artificially acquired, for its ultimate effects penetrate much deeper than the surface, and end by changing, or helping to change the intimate organizations of the body itself, entirely and eventually for the better. It is a regimen which, like that undergone by the jockey for the purpose of racing, or by the prize-fighter, alters the form and texture, both inward and outward, of his frame, and reduces it to the precise weight and measure required for his service. It is, in the literal sense of the word, a recre-

* There is one regulation of the health which, simple as it may seem to be, is of the utmost importance in the treatment of children liable to cerebral excitement, as well as to maniacal or idiotic symptoms:—it is, that they should never be allowed to eat between their meals. Insignificant as this direction may sound, it is, notwithstanding, one of those items the importance of which can be recognised and fully appreciated only by a practical man, for nothing irritates the temper so much, and helps to keep up a continual fret upon the mind and spirits, as an irregularity of this description. Nothing should be taken between meals, not even a glass of water, and the meals should punctually return at their stated hours.

ation or renovation which may be rendered so complete as to surpass the distant hopes that the serious nature of the malady had at first reasonably led us to entertain.

The influence of diet, climate, and locality over the character, dispositions, and morals of a people, both nationally and individually, is a doctrine that has received the countenance and support of some of the greatest names in science and literature, such as Hippocrates among the ancients, and Montesquieu in much more recent times.* That so inconstant, self-willed, and independent a being as man should, like the inferior animals, be subjected to the perpetual modifications arising from food, temperature, air, and soil, might at the first glance seem to be a bold proposition. But, nevertheless, so it is; and, within the limits of certain restrictions, there can be no question that man, both bodily and mentally, is, with all his intellectual ascendancy, the helpless creature of telluric and sidereal agencies. In many points, the doctrine of Hippocrates, concerning the varieties of mankind, agrees with that of M. Geoffry Saint-Hilaire upon the varieties of domestic animals; and if, on the one side, according to the French naturalist, the number and degrees of varieties among animals expresses the number and degrees of the different influences to which they are submitted; so, on the other, according to the Greek physician, the varieties of people represent the varieties of soil and climate, while the resemblances between the individuals of the same nation prove that these same individuals are exposed, upon an extensive scale, to the same influences, whether we regard them as the results of a semibarbarous state, like that of the ancient Scythians or modern natives of New Zealand, or suppose them to be the effect of castes such as those mentioned of Egypt more than three thousand years ago, or such as we may discover for ourselves among the various groups that compose our Anglo-Indian empire at the present day.

We have, in the preceding observations, taken but a cursory view of the all important subject, the *education of children predisposed to mental derangement;* at present this field of inquiry is untrod—thousands of children, with a clear and obvious predisposition to insanity, are left without the advantages of that education of the moral, intellectual, and physical faculties, which alone can save them from the fearful abyss, into which, at a future period, they must be hurled, unless something is done to avert so direful a calamity! In many of these cases, where the nervous system is in a precocious state of development, and the unnatural amount of intellectual capacity and vital energy developed in early life clearly indicate the existence of a latent tendency to affections of the cerebral apparatus, nothing literally is done to quell the impending storm, or adapt the vessel for the approaching hurricane;—the mother and the father, delighted with the precocity evinced by the darling child—the infant prodigy—instead of keeping down these unnatural and unhealthy manifestations of nervous and mental vigour (the sure harbingers of terrible disease!) do their best to encourage the excited brain and fragile mind to the exercise of its *utmost power*, until the poor creature sinks prematurely into the grave, the victim of water in the brain, or chronic

* Œuvres complètes d'Hippocrate, Traduction Nouvelle, par E. Littré, tome ii. pp. 3—5. Paris, 1840.

inflammation of the encephalon; or lives only to linger out a painful existence, at an advanced period of life in a state of positive imbecility, or inmate of a lunatic asylum. Mothers! fathers! listen to the voice of experience. Remember that the precocious child is often like a meteor—it flashes in all its brilliant effulgence for a few minutes above us, and then expires. Believe us, when we say, that the seeds of fatal, incurable, melancholy disease of the brain and mind are often the consequences of the mistaken fondness and excessive indulgence of those who ought to be the last to bring about such sad results!

The consumptive, the scrofulous, the gouty diatheses, are marked in the outward lineaments of the human frame, and the practised eye of the physician can generally predicate with accuracy the possibility of such affections being developed at certain ages, provided the constitution is subjected to agencies known to excite into actual development these diseases.

In the same manner the *maniacal diatheses* is easily detected by the observant and experienced physician, and by the use of all devised means the brain and its appendages may be preserved intact and free from any serious affections. Our object in this paper is more to point out the importance of education under these circumstances, than to lay down any minute and specific rules for the guidance of those to whose care such cases may be trusted. At some other time we purpose fully discussing this subject in all its important ramifications.

